# Editorial: Non-invasive brain stimulation techniques in neurological and neuropsychiatric disorders: Physiological and molecular evidence

**DOI:** 10.3389/fnsys.2023.1128205

**Published:** 2023-02-06

**Authors:** Marco Cambiaghi, Marika Cordaro, Silvia Dossena, Salvatore Cuzzocrea, Mario Buffelli

**Affiliations:** ^1^Department of Neuroscience, Biomedicine and Movement Sciences, University of Verona, Verona, Italy; ^2^Department of Biomedical and Dental Sciences and Morphofunctional Imaging, University of Messina, Messina, Italy; ^3^Institute of Pharmacology and Toxicology, Paracelsus Medical University, Salzburg, Austria; ^4^Department of Chemical, Biological, Pharmaceutical and Environmental Sciences, University of Messina, Messina, Italy

**Keywords:** brain stimulation, neuromodulation, vagal nerve stimulation (VNS), neuroenhancement (NE), prefrontal cortex

The idea of treating brain disorders with non-invasive stimulation (NIBS) techniques dates back several centuries and nowadays is among the main therapeutical prospects of modern neurology and psychiatry (Peruzzotti-Jametti et al., 2013; Rossini et al., 2015; Cambiaghi and Sconocchia, 2018). Non-invasive stimulation technologies include a wide range of options, ranging from transcranial magnetic stimulation (TMS), different transcranial electrical stimulation (tES) approaches, vagal nerve stimulation (VNS), and focused ultrasound stimulation (FUS). This set of techniques has been extensively used for studying the CNS physiology and the functional role of specific brain structures, as well as for a modern therapeutical approach for several brain diseases.

The present Research Topic collects five manuscripts highlighting as many different aspects within this heterogeneous field of study, ranging from the use of NIBS techniques in physiological conditions, like human brain augmentation or the modulation of neural plasticity and behavior in mice, up to the treatment of neuropathological conditions, such as Alzheimer disease or persistent posttraumatic symptoms following mild-moderate traumatic brain injury.

Vagal nerve stimulation based on implanted devices (iVNS), firstly approved by FDA in 1997 for treating epilepsy and later for depression, suffers from some safety issues that paved the way for the development of transcutaneous VNS (tVNS), that instead can be applied through locations on the ear (auricular) or the neck (cervical) (Butt et al., 2020). In their mini-review, Vargas-Caballero et al. discuss the rationale for tVNS being a reliable therapy for treating cognitive symptoms characterizing early stages of Alzheimer Disease (AD). Among the different possible mechanisms of action, the authors focus their attention on the activation of the locus coeruleus (LC), which results in catecholamines release in the hippocampus and neocortex, with a subsequent enhancement of synaptic plasticity and reduction of inflammatory signaling. Indeed, loss of noradrenergic LC projection neurons that mediate attention, memory, and arousal has been observed in AD patients, with the number of this noradrenergic neurons also associated with increased postmortem neuropathological findings (Kelly et al., 2017).

In recent years, NIBS has also been associated with the possibility to increase human mental capacities, namely neuroenhancement, a challenge with a long tradition in science (Antal et al., 2022). Most cognitive enhancement methods can be classified as biochemical, physical, or behavioral interventions and include the use of traditional medicines or pharmaceuticals, lifestyle modifications, as well as non-invasive and invasive brain stimulation techniques, respectively. In their review article, Jangwan et al. offer an overview on the actual and possible applications, advantages and shortcomings of brain augmentation technologies. Brain augmentation, also referred to as cognitive enhancement, includes approaches from different disciplines aimed at the improvement of brain performance in both healthy people and patients suffering from neurological disabilities. In addition to enhancing memory and cognition in healthy people, current applications of non-invasive brain augmentation techniques include stroke and vascular dementia, epilepsy, attention-deficit hyperactivity disorder (ADHD), Tourette's syndrome, autism spectrum disorders (ASD), depression, and schizophrenia. Moreover, the use of prosthetics and brain-computer interfaces plays a major role in rehabilitation. However, the authors highlight that the use of brain augmentation techniques to promote brain function beyond the normal health status opens important ethical issues. Thus, they conclude that the safety and efficacy of brain stimulation techniques for the treatment of neurological and neuropsychiatric disorders deserves to be further explored by well-designed clinical trials in the future.

Different neurological and neuropsychiatric disorders are associated with abnormal neural oscillations within the gamma-band (30–80 Hz), and altered neural synchronization plays a key role in many cortico-cortical processing (Singer, 1999). Different NIBS techniques, especially transcranial altering current stimulation (tACS) and repetitive transcranial magnetic stimulation (rTMS), are able to modulate brain oscillations in both healthy and clinical populations (Rossi et al., 2022). In a mini-review article Griskova-Bulanova et al. point out that, so far, only a limited amount of research is devoted to analyzing the impact of neuromodulation on gamma-range auditory steady-state responses (ASSRs). The ASSRs are scalp-recorded signals reflecting the capability of the auditory system to follow the timing patterns of acoustic stimuli that stay constant over time, and they are considered translational EEG biomarker of the brain's ability to process sensory information (O'Donnell et al., 2013). Indeed, ASSRs reveal the functional integrity of the neural system and the excitation/inhibition balance, as they are based on both NMDA and GABA systems, and alterations of 40-Hz ASSRs are often observed in schizophrenia (Tada et al., 2020). NIBS has the potential for being considered a strong tool to modulate the neural circuits underlying ASSRs.

In their article, Quinn et al. investigate the use of anodal transcranial direct current stimulation (tDCS) as a therapeutical tool to ameliorate pathophysiological processes contributing to persistent posttraumatic symptoms (PPS) after a mild-moderate traumatic brain injury (mmTBI). In a cohort of 34 subjects with mmTBI, 10 anodal tDCS sessions over the left dorsolateral prefrontal cortex (DLPFC) resulted in an improved working memory reaction time after 1 month. Moreover, the authors observed that the reaction time improvement correlated with a reduced functional connectivity between the right DLPFC and the left anterior insula, with hyperconnectivity suggested to be a chronic compensatory state (Iraji et al., 2016). Of note, the Neurobehavioral Symptom Inventory revealed a significant effect of time on emotional symptoms, known to be an important target of NIBS of the left DLPFC (Mondino et al., 2015).

Emotional behavior is managed in the paper by Cambiaghi et al., where the authors explore the effects of 15 Hz rTMS over the prefrontal cortex (PFC) in wild-type mice, focusing on structural neuroplasticity and behavior. Though in humans rTMS over PFC is known to modulate emotions, cognition, and depressive symptoms, the underlying mechanisms of action are still far from being clear. The authors report here that, after a 5-days high-frequency rTMS protocol, mice displayed a decreased depressive-like behavior, associated with increased spine density in layer II/III and layer V of PFC apical and basal dendrites. This increase in the number of spines goes in parallel with an increased complexity in the neural arborization within the same region, suggesting a structural remodeling of the medial PFC. This area is known to be key for the modulation of various abnormal behaviors (Chini and Hanganu-Opatz, 2021), and a better knowledge of NIBS effects would be of great importance. The same authors had already shown that the same stimulation protocol had a similar plastic change on the motor cortex, though with a lower impact (Cambiaghi et al., 2021), while low-frequency rTMS had similar, but more modest, behavioral outcomes (Cambiaghi et al., 2020b). Moreover, they observed that also prefrontal tDCS in the mouse is able to modulate serotonergic activity, one of the main systems involved in the pathophysiology and psychopharmacology of depression (Cambiaghi et al., 2020a).

This collection of articles highlights the significance of a better understanding of the mechanisms underlying the effects associated to NIBS techniques, for not only neuroscientists but also neurologists, psychiatrists and physiotherapists.

## Author contributions

MCa wrote the first draft. MCo, SD, SC, and MB provided critical comments and editorial suggestions for revisions. All the authors agreed on the submitted version.

## References

[B1] Antal A. Luber B. Brem A. K. Bikson M. Brunoni A. R. Cohen Kadosh R.. (2022). Non-invasive brain stimulation and neuroenhancement. Clin. Neurophysiol. Pract. 7, 146–165. 10.1016/j.cnp.2022.05.002 35734582 PMC9207555

[B2] Butt M. F. Albusoda A. Farmer A. D. Aziz Q. (2020). The anatomical basis for transcutaneous auricular vagus nerve stimulation. J. Anat. 236, 588–611. 10.1111/joa.13122 31742681 PMC7083568

[B3] Cambiaghi M. Buffelli M. Masin L. Valtorta F. Comai S. (2020a). Transcranial direct current stimulation of the mouse prefrontal cortex modulates serotonergic neural activity of the dorsal raphe nucleus. Brain Stimul. 13, 548–550. 10.1016/j.brs.2020.01.012 32289674

[B4] Cambiaghi M. Cherchi L. Masin L. Infortuna C. Briski N. Caviasco C.. (2021). High-frequency repetitive transcranial magnetic stimulation enhances layer II/III morphological dendritic plasticity in mouse primary motor cortex. Behav. Brain Res. 410, 113352. 10.1016/j.bbr.2021.113352 33979657

[B5] Cambiaghi M. Crupi R. Bautista E. L. Elsamadisi A. Malik W. Pozdniakova H.. (2020b). The effects of 1-Hz rTMS on emotional behavior and dendritic complexity of mature and newly generated dentate gyrus neurons in male mice. Int. J. Environ. Res. Public Health 17, 4074. 10.3390/ijerph17114074 32521613 PMC7312937

[B6] Cambiaghi M. Sconocchia S. (2018). Scribonius Largus (probably before 1CE-after 48CE). J. Neurol. 265, 2466–2468. 10.1007/s00415-018-8739-5 29356976

[B7] Chini M. Hanganu-Opatz I. L. (2021). Prefrontal cortex development in health and disease: lessons from rodents and humans. Trends Neurosci. 44, 227–240. 10.1016/j.tins.2020.10.017 33246578

[B8] Iraji A. Chen H. Wiseman N. Welch R. D. O'Neil B. J. Haacke E. M.. (2016). Compensation through functional hyperconnectivity: a longitudinal connectome assessment of mild traumatic brain injury. Neural Plast. 2016, 4072402. 10.1155/2016/4072402 26819765 PMC4706919

[B9] Kelly S. C. He B. Perez S. E. Ginsberg S. D. Mufson E. J. Counts S. E.. (2017). Locus coeruleus cellular and molecular pathology during the progression of Alzheimer's disease. Acta Neuropathol. Commun. 5, 8. 10.1186/s40478-017-0411-2 28109312 PMC5251221

[B10] Mondino M. Thiffault F. Fecteau S. (2015). Does non-invasive brain stimulation applied over the dorsolateral prefrontal cortex non-specifically influence mood and emotional processing in healthy individuals? Front. Cell. Neurosci. 9, 399. 10.3389/fncel.2015.00399 26528131 PMC4604238

[B11] O'Donnell B. F. Vohs J. L. Krishnan G. P. Rass O. Hetrick W. P. Morzorati S. L.. (2013). The auditory steady-state response (ASSR): a translational biomarker for schizophrenia. Suppl. Clin. Neurophysiol. 62, 101–12. 10.1016/B978-0-7020-5307-8.00006-5 24053034 PMC4959266

[B12] Peruzzotti-Jametti L. Bacigaluppi M. Sandrone S. Cambiaghi M. (2013). Emerging subspecialties in neurology: transcranial stimulation. Neurology 80, e33–e35. 10.1212/WNL.0b013e3182833d74 23339212

[B13] Rossi S. Santarnecchi E. Feurra M. (2022). Noninvasive brain stimulation and brain oscillations. Handb. Clin. Neurol. 184, 239–247. 10.1016/B978-0-12-819410-2.00013-8 35034738

[B14] Rossini P. M. Burke D. Chen R. Cohen L. G. Daskalakis Z. Di Iorio R.. (2015). Non-invasive electrical and magnetic stimulation of the brain, spinal cord, roots and peripheral nerves: basic principles and procedures for routine clinical and research application. An updated report from an I.F.C.N. Committee. Clin. Neurophysiol. 126, 1071–1107. 10.1016/j.clinph.2015.02.001 25797650 PMC6350257

[B15] Singer W. (1999). Neuronal synchrony: a versatile code for the definition of relations? Neuron 24, 49–65, 111–125. 10.1016/S0896-6273(00)80821-1 10677026

[B16] Tada M. Kirihara K. Koshiyama D. Fujioka M. Usui K. Uka T.. (2020). Gamma-band auditory steady-state response as a neurophysiological marker for excitation and inhibition balance: a review for understanding schizophrenia and other neuropsychiatric disorders. Clin. EEG Neurosci. 51, 234–243 10.1177/1550059419868872 31402699

